# Diagnostic Difficulties in Evaluation of Neck Masses – Idiopathic Lymph Node Infarction

**DOI:** 10.25122/jml-2018-0047

**Published:** 2018

**Authors:** Imre Subicz, Balázs Sztanó, Géza Krasznai, András Vörös, László Tiszlavicz, Rezsö Borkó, László Rovó

**Affiliations:** 1. Department of Oto-Rhino-Laryngology, Jász-Nagykun-Szolnok County Hospital, Szolnok, Hungary; 2. Department of Oto-Rhino-Laryngology Head and Neck Surgery, University of Szeged, Hungary; 3. Department of Pathology, Jász-Nagykun-Szolnok County Hospital, Szolnok, Hungary; 4. Department of Pathology, University of Szeged, Hungary

**Keywords:** neck mass, lymph node infarction, fine needle aspiration cytology

## Abstract

Palpable neck masses are often the only signs of patients visiting their ENT specialists. Lymphadenopathy may be a primary or secondary manifestation of numerous benign and malignant disorders. The medical history, physical examination, imaging and pathological examination may help to set the appropriate diagnosis. Lymph node infarction is a very rare entity among the various pathologies involving the lymph nodes. We hereby present three cases, in which infarction was the only symptom, no associated condition occurred.

## Introduction

Differential diagnosis and treatment of a neck mass is a frequently encountered problem in ENT practice. Several different congenital, inflammatory and benign or malignant tumors may cause a palpable neck lump [1, 2, 3]. A relevant medical history should be obtained and a complete examination of the oral cavity, pharynx, larynx, rhinopharynx and nasal cavities is mandatory and should be performed. Modern imaging, ultrasonography, computed tomography and magnetic resonance imaging play an essential role [4, 5]. Laboratory investigations and viral serology are performed as well. Solid neck masses without obvious etiology should be investigated using fine needle aspiration cytology, as it has a good diagnostic reliability of 85% and a specificity of 99% [6]. In uncertain cases, an excisional biopsy may be necessary.

This algorithm usually helps to make the diagnosis, but sometimes ENT specialists must face a great differential diagnostic challenge. In this article, the authors present three neck lymph node infarction cases, in which no associated malignant or benign disorder could be discovered.

### Case 1

P.F., a 75-year-old man presented with a left side submandibular mass, 4 cm in diameter. He had noticed the painless mass 6 months earlier, which had started to grow. The patient did not have any swallowing or breathing problems. Micolaryngoscopy revealed no malignant tumor in the head-neck region. Removal was performed and under the platysma muscle, a solid, encapsulated mass was found. After surgery, the patient lived without any recurrence for 8 years.

Histology described that coagulative necrosis replaced the original histologic structures, and it was surrounded by a thick, artificially damaged fibrotic capsule. Residual lymphoid cell clusters could be seen only within the capsule, referring to the previous lymphoid tissue. Signs of previous bleeding could be detected with intra- and subcapsular accumulation of decompensated red blood cells and haemosiderin-laden macrophages. The capsule showed reactive, reparative changes with macrophages, plasma cells surrounded by fibroblast accumulation and newly formed capillaries. Perinodal connective tissue contained some small vessels showing signs of productive vasculitis with endothelial proliferation, and occasionally lumen obstruction. The wall of obstructed vessels was infiltrated by mononuclear cells. Cross sections of four further reactive lymph nodes could be detected within the surrounded fat tissue with reactive follicular hyperplasia and sinus histiocytosis. The diagnosis was lymph node infarction with negative surrounding lymph nodes and obliterative vasculitis nearby the infarction. No signs of malignancy could be detected.

### Case 2

**Figure 1:**
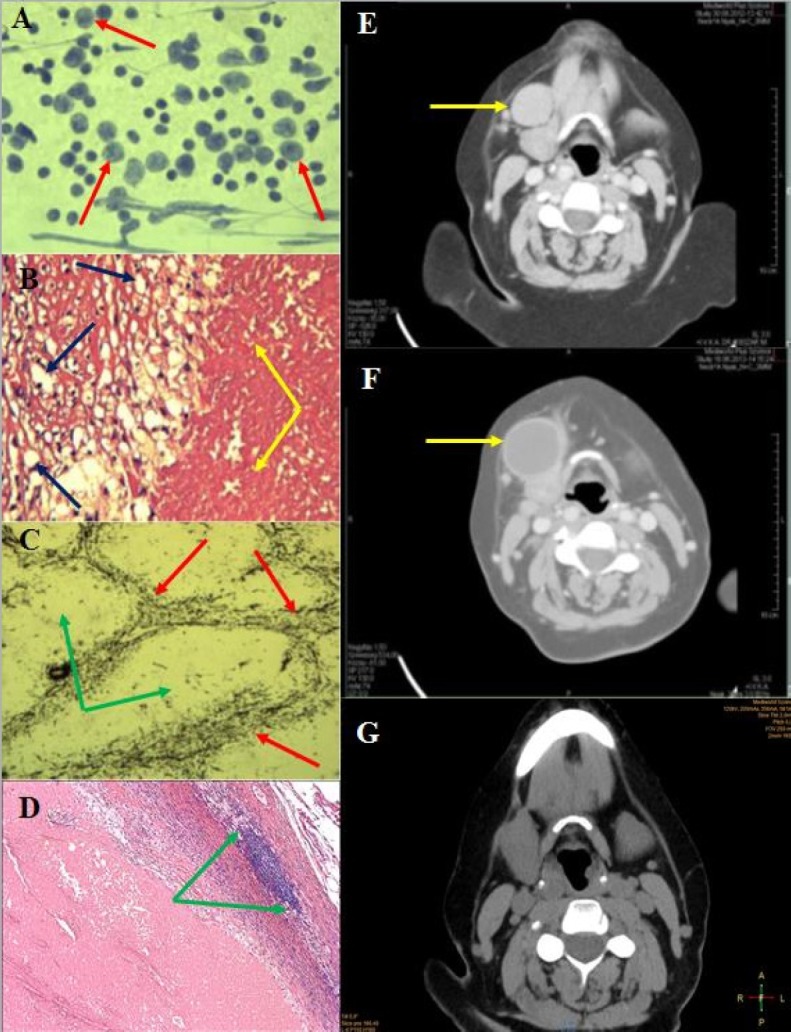
Idiopathic lymph node infarction in a 62-year-old female A: Fine needle aspiration cytology: various sized and shaped lymphoid elements (arrow); B: Histology (HE staining): inhomogeneous necrosis (yellow arrow), fibrous capsule with macrophages (blue arrow); C: Histology (silver impregnation): fibrotic structure of the lymph node is maintained, so the follicular structures are visible within the necrotic area (red arrow); the centers of the follicles do not contain any fibrotic background (green arrow); D: Histology (HE staining): residual lymphoid tissue (between green arrows), necrosis with a thick fibrous capsule; E: Neck CT (06.2012): A solid enlarged lymph node near the right submandibular gland; F: Neck CT (05.2013): arrow shows solid cystic resistance; G: Neck CT (01.2016): no pathologic lymph node can be detected.

V.A., a 62-year-old female, presented with a 4 cm in diameter, mobile submandibular mass on the right side in June 2012. Physical examination revealed no further abnormality. Neck CT showed a submandibular mass located just near the submandibular gland (Figure 1E), and the suggested diagnosis was enlarged lymph node or connective tissue tumor. Following the diagnosis, the patient became unreachable and was only encountered again one year later (in May 2013) with similar symptoms, albeit with complaints of a lump enlargement. Her general condition was appropriate; she did not exhibit any weight loss, pain, swallowing or breathing disorders. Blood tests’ values were normal, chest X-ray, abdominal ultrasound did not display any concerning images. Fine needle aspiration cytology suggested the diagnosis of a metastasis of a high-grade malignant tumor or less likely a neuroendocrine tumor (Figure 1A). Repeated neck CT found a right submandibular mass with surrounding lymph nodes, supposedly an inflammatory cyst (Figure 1F). Panendoscopy revealed no head-neck primary tumor. Finally, in November 2013 the lump along with lymph nodes was removed.

Histology showed a cyst-like lesion which contained necrotic tissue mass with peripheral infiltration of histiocytes and macrophages (Figure 1B). Immunohistochemistry of the necrotic area showed pan-CK negativity. Diffused LCA, focal CD-3 and CD-20 positivity could be detected (Figure 1C). The necrotic tissue was surrounded by a thick fibrous capsule (Figure 1D). Additionally, some reactive lymph nodes without any signs of malignancy were found. The histologic diagnosis was enlarged necrotic lymph node (lymph node infarction). Due to extensive necrosis, lymphoproliferative disease or any other malignancy including metastatic tumors could not be definitely excluded.

During the 3-year follow up period, the patient was tumor free, and no lymph node enlargement occurred again. Routine neck CT showed no abnormality in January 2016 (Figure 1D).

### Case 3

**Figure 2:**
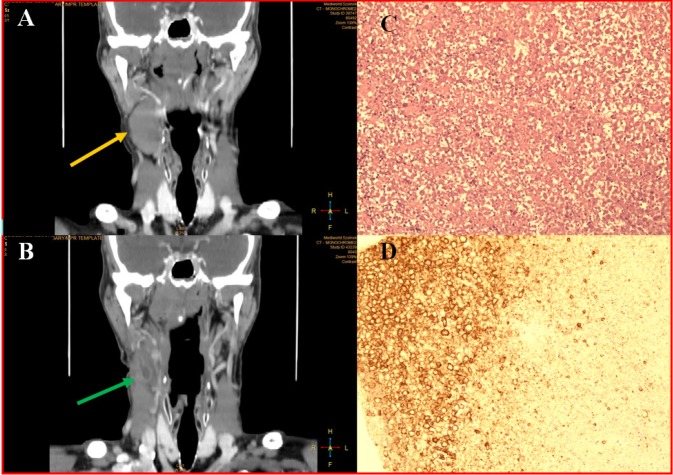
Idiopathic lymph node infarction in a 70-year-old man A: Neck CT (10.2015): homogenous, hypodense 33x25mm mass in right neck Level II (yellow arrow); B: Neck CT (05.2016): mass decreased in size (17mm) and became heterogenous (green arrow) C: Histology (HE staining): inhomogeneous necrosis, the contours of the cells can be seen without any nuclear staining; D: Histology (IHC): necrosis surrounded by CD20 positive residual lymphatic tissue.

SZ.F., a 70 years old male presented with a 4 cm lump in the neck (Level II). There was no significant abnormality in blood tests. Fine needle aspiration cytology showed it was a lymphoproliferative disorder, and hematological examination was suggested. In the neck CT scans a 33x25 mm hypodense, homogenous mass was found, which could be a lymph node (Figure 2A). In the surrounding area, there were other smaller lymph nodes as well. No primary tumor or hematological disorder could be explored. The lump started to grow and became painful and an open surgical biopsy was conducted.

Histology found necrotic tissue with a fibrotic capsule. The capsule contained Vimentin-positive connective tissue components and vessels. Some fragments of skeletal muscle embedded in the capsule could also be detected and the necrotic area seemed to be necrotic lymphoid tissue. Silver impregnation showed a residual reticular sinusoidal structure. CD-20, CD-68 immunohistochemistry in the necrotic area indicated some histiocytes and residual lymphoid cells and the Ki-67 reaction shows a 10% proliferation index of CD-20 positive lymphocytes (Figure 2C, 2D). The diagnosis was subtotal lymph node infarction without any sign of malignancy.

After a few months, the lump decreased in size, but a fistula occurred in the incision area. Routine CT scan only showed a 17 mm in diameter cystic mass (Figure 2B), which was finally removed in toto. The final histologic diagnosis was the same, a lymph node infarction without any sign of malignancy.

## Discussion

Lymph node infarction is a very infrequent phenomenon reported in the literature in which an enlarged lymph node shows coagulative necrosis of at least a large confluent area of the nodal tissues [7, 8]. Lymph nodes are well vascularised and therefore infarction is uncommon. Infarcted lymph nodes are often located in the head and neck region, for example in Jiang’s study of 35 cases, 40% occurred in the neck [9].

The type of coagulative necrosis indicates the ischemic nature of the necrosis. In superficial lymph nodes, arteries and veins enter the node through the hilar region. This vascular distribution explains the sparing of subcapsular lymphoid tissue in lymph node infarction. In contrast, deep lymph nodes which lack a hilum show more extensive necrosis [8].

Lymph node infarction is associated with various non-neoplastic and neoplastic conditions. In their literature review, Jiang et al. presented 33 papers dealing with 256 lymph node infarction cases, 175 malignant (68%) and 81 non-malignant (32%) cases [9]. Neoplastic lesions most commonly associated with infarction are malignant lymphoma and metastatic malignancy [9, 10]. According to that study, 90% (159/175) of malignant cases were associated with haematolymphoid malignancy and 10% (16/175) with metastatic tumors [9]. Massive infarction of the lymph node should raise the suspicion of lymphoma and must be carefully investigated [11]. Based on Jiang’s immunohistochemistry diffuse, large B-cell lymphoma, follicular lymphoma, and anaplastic large cell lymphoma were the most common types [9, 12].

Non-neoplastic causes are polyarteritis nodosa, viral infections (infectious mononucleosis, parvovirus B19), thrombosis, cholesterol atheromatous embolism, mononeuritis multiplex, intestinal volvulus, disseminated intravascular coagulation, mechanical trauma, medicine-induced [13–18]. Iatrogenic manipulations, such as intramuscular gold injections for the treatment of rheumatoid arthritis, post-mediastinoscopy procedures or heart-lung transplantation, increase the risk [19]. Fine needle aspiration cytology can also lead to infarction [20, 21], but it is not frequent, in a large series of 230 aspirated and later excised lymph nodes it was found in 4% [22]. Finally, as ‘exclusion diagnosis’, it may be idiopathic.

The presented three cases were idiopathic. Laboratory tests, imaging, and fine needle aspiration cytology revealed no associated disorder. Although in Maurer’ study 16% (6/37) and Jiang’s study 42% (3/7) apparently ’benign’ lymph node infarction showed the manifestation of malignant lymphoma in the follow-up period, fortunately, none of the authors’ cases had any malignancy within the following 3–9 years.

## Conclusion

Although lymph node infarctions are associated in the vast majority of cases with malignant lymphomas and metastatic malignancies, sometimes they pose a great differential diagnostic challenge. In our cases where idiopathic infarcted lymph nodes occurred, no associated disorder could be recognized during several years of follow-up, and after their removal, they did not reoccur.

## Conflict of Interest

The authors confirm that there are no conflicts of interest.

**Ethical approval:** All procedures performed in the study involving human participants were in accordance with the ethical standards of the University of Szeged, Hungary.

Informed consent was obtained from all individual participants included in the study.
